# Anti-Tumor Activity of Cembranoid-Type Diterpenes Isolated from *Nicotiana tabacum* L.

**DOI:** 10.3390/biom9020045

**Published:** 2019-01-28

**Authors:** Xiao-Long Yuan, Xin-Xin Mao, Yong-Mei Du, Pei-Zhen Yan, Xiao-Dong Hou, Zhong-Feng Zhang

**Affiliations:** Tobacco Research Institute of Chinese Academy of Agricultural Sciences, Qingdao 266101, China; rayrock@126.com (X.-L.Y.); maoxinxincaas@163.com (X.-X.M.); duyongmei@caas.cn (Y.-M.D.); yanpeizhen1213@163.com (P.-Z.Y.)

**Keywords:** antitumor activity, α-cembratriene-diol, cytological observation, *Nicotiana tabacum* L., transcriptome

## Abstract

Recently, the incidence of hepatocellular carcinoma has increased worldwide. Cembranoid-type diterpenes (CBDs) from tobacco exhibit good antimicrobial, antitumor, and neuroprotective activities. Therefore, in this study, we isolated CBDs from *Nicotiana tabacum* L. and evaluated their antitumor activity against hepatoma cell lines. Particularly, the anti-tumor activity of α-2,7,11-cyprotermine-4,6-diol (α-CBD) was investigated against HepG2, SMMC-7721, and HL-7702 cells. The MTT assay revealed that α-CBD reduced the formation of cell clones and inhibited the proliferation of hepatocellular carcinoma cells. Morphological observations showed that α-CBD altered cell morphology and membrane permeability before inducing apoptosis. To further explore the antitumor mechanism of α-CBD, flow cytometry and transcriptome analysis were performed using HepG2 cells. The results showed that the number of HepG2 cells increased from 10.4% to 29.8%, indicating that α-CBD inhibits the proliferation of hepatocellular carcinoma cells in the S phase. The gene expression analysis of HepG2 cells treated with α-CBD showed 3068 genes with altered expression, among which 1289 were upregulated and 1779 were downregulated. Apoptosis induced by these differentially expressed genes might be mediated by the p53-PUMA, PI3K-Akt, and IL-1-NF-κB-IAP pathways. Comprehensively, our study shows that α-CBD isolated from *N. tabacum* L. can be potentially used as a natural antitumor agent.

## 1. Introduction

Malignant tumors are a major public health issue worldwide. Currently, cancer has become the second-leading cause of death, and is expected to surpass the current leading cause of death-heart diseases-in the future [1]. Experts from the National Center for Health Statistics have predicted that by 2030 cancer-related deaths could increase globally by as much as 80% [2]. Recent studies have mainly focused on surgery, radiotherapy, chemotherapy, and biotherapy, and in exploring possible approaches to improve these methods [3]. Surgery has been proven to be the most effective therapy; however, only a small percentage of patients with a malignant tumor have the chance of surgery and survival. Moreover, the recurrence rate of malignant tumors is still at a relatively high level [4]. Therefore, it is important to explore effective antitumor therapies.

Traditional Chinese Medicines are considered as an alternative source of antineoplastic drugs [5]. As China is rich in natural resources, several medicinal plants are widely used for treating various ailments and antineoplastic drugs [6]. Examples of useful active compounds contained in plants include sesquiterpene, lactones, diterpenes, quassinoids, triterpenes, alkaloids, quinones, diamides, coumarins, flavonoids, lignans, macrolides, polyacetylenes, polyphenols, and styrylpyrones. Their derivatives and analogs also play an important role as antineoplastic drugs [7]. Prior to exploring the function of these active compounds, appropriate isolation is required to ensure that their function can be detected by multiple methods. In addition, we need to understand their antitumor mechanisms.

As a mode of cell death, apoptosis plays an important role in the origin and development of tumor, and thus, in antitumor drug research [8]. Anti-tumor drugs can inhibit cell growth and induce apoptosis of tumor cells by interfering with their growth, metabolism, proliferation and cell cycle [9,10,11,12]. In addition, there is also accumulating evidence that the efficiency of anti-tumor agents is related to gene regulation and related pathways [13,14,15].

Recently, active ingredients from plants that induce apoptosis of tumor cells have attracted attention as antitumor agents. Cembranoid-type diterpenes (CBDs) were first identified in tobacco and pines [16,17]. It has been shown that CBDs isolated from tobacco have exhibited good antimicrobial, antitumor, and neuroprotective activities [18,19]. However, studies on the mechanisms underlying the anticancer effects of CBDs against hepatocellular carcinoma cells are required. Hepatocellular carcinoma is the second most common cancer worldwide, and its incidence rate is gradually increasing [20]. The inhibitory effect of CBDs on hepatoma cell lines has been rarely reported. Therefore, to investigate the antitumor activity of CBDs from tobacco and enable the use of tobacco resources, α-2,7,11-cyprotermine-4,6-diol (α-CBD) was evaluated. Through this study, we hope to make use of abundant tobacco resources, develop beneficial substances from tobacco, and reduce the cost of extraction and use of active substances from tobacco. We also hope to unveil more natural antitumor substances from tobacco and understand their antitumor mechanism. This will enable their application in clinical therapy in the future.

## 2. Material and Methods

### 2.1. Plant Identification

The plant *Nicotiana tabacum* L. V2 (Family: Solanaceae) was collected in China Tobacco Germplasm Platform (Qingdao, China) on June 2016. The plant material was identified by the Prof. Xing-Wei Zhang (Tobacco Germplasm Platform, Qingdao, China). A voucher specimen (TGPC 16-6-05) has been deposited in our laboratory. In addition, these strains were then stored in the Tobacco Research Institute of Chinese Academy of Agricultural Sciences for the following studies. Inflorescences of the *N. tabacum* are sparsely branched panicles. In this study, the inflorescences of *N. tabacum*. were used for the α-CBD isolation.

### 2.2. Extraction and Isolation of α-CBD from Nicotiana tabacum L.

The inflorescence of *N. tabacum*. was immersed in dichloromethane at 25 °C three times, with each immersion lasting 1–2 s. The extracted solutions were pooled and filtered. Furthermore, 10 g of the crude extract was dissolved and washed three times with 300 mL of 70% ethanol. The supernatants were collected and concentrated to 300 mL. Subsequently, 300 mL of petroleum ether was added to the solution and extraction was performed thrice. The petroleum ether layer was combined and the crude extract of pale-yellow sisonide diterpene was concentrated. One gram of Nishimatsu alkanes diterpene crude extract was subjected to silica gel column chromatography. The chromatography samples (5:1, *v*/*v*) and petroleum ether/ethyl acetate (3:1, *v*/*v*) were successively added to the chromatographic column (7.8 mm id × 30 cm, Waters, Milford, MA, USA) for elution. We collected and concentrated the second fraction. Colorless oily monomer compounds were obtained by silica gel column chromatography. The monomer compounds were further confirmed by nuclear magnetic resonance.

### 2.3. Cell Culture and Treatment with α-CBD

The cell lines used (HepG2, SMMC-7721, and HL-7702) were purchased from the Chinese Academy of Sciences Committee on Type Culture Collection Cell Bank (Shanghai, China). HepG2 and SMMC-7721 cells were cultured in MEM (Gibco AG, Basel, Switzerland), whereas HL-7702 cells were cultured in RPMI-1640 supplemented with 10%fetal bovine serum, 1% penicillin, and 1% streptomycin. The medium was replaced every 2–3 days. All the cells were cultured in a humidified atmosphere with 5% CO_2_ at 37 °C. Prior to treatment with α-CBD, the cell density was adjusted to 5 × 10^4^ cells/mL, and the cell suspension was poured into 96-well plates. α-CBD was extracted with a purity of more than 95%. The obtained α-CBD was dissolved in 5% DMSO.

### 2.4. Detection of Viability and Clone Formation Ability of Hepatocellular Carcinoma Cells

To detect the inhibitory effect of α-CBD on the proliferation of carcinoma cells, α-CBD was added to the three cell line cultures to final concentrations of 80, 40, 20, 10, 5, 2.5, and 1.25 mg/L and incubated for 24, 48, and 72 h. The control groups were treated with culture media. Three replications were performed for each concentration. Twenty microliters of (5 mg/mL) MTT solutions were added to each sample, 4 h before the completion of experiment. After incubation, the culture medium was carefully aspirated, and 150 mL of DMSO solution was added to each well. Subsequently, the 96-well plates were shaken at a low speed on an oscillator for 10 min. The absorbance of the samples was measured using a microplate spectrophotometer reader (Multiskan GO, Thermo Scientific, Waltham, MA, USA) at a wavelength of 570 nm to detect the growth rate of each sample. Meanwhile, the ability of hepatocellular cells to form clones was detected. After the treatment with α-CBD, the cells were digested with 0.25% trypsin, diluted to 100–1000 cells/mL, and inoculated in a culture dish for another two weeks. The cells were then washed twice with phosphate-buffered saline (PBS) and 10 mL of 0.01 g/mL Giemsa dye was added for staining. The colonies were photographed and counted. The results were analyzed using SPSS 21.0 software package (Chicago, IL, USA)

### 2.5. Cellular Morphology Observation

The effects of α-CBD on cell morphology were studied in HepG2 and SMMC-7721 cells. Briefly, the cells were exposed to 20 mg/L α-CBD for 24, 48, and 72 h followed by harvest as mentioned above. The cells were then stained with Giemsa and observed under a microscope. Meanwhile, the morphology of the two cell lines was observed using Hoechst 33258 and acridine orange/ethidium bromide (AO/EB) staining, by fluorescence microscopy (Ti Eclipse, Nikon, Tokyo, Japan).

### 2.6. Flow Cytometry Analysis

To detect the effect of α-CBD on cell cycle, HepG2 cells treated with 20 mg/L α-CBD for 24, 48, and 72 h were collected at a concentration of 1 × 10^6^ cells/mL. The cells were then stained with 20 μg/mL propidium iodide (PI) solution (containing 0.1% TritonX-100 and 100 μg/mL RNase A). A FACScan flow cytometer (BD Biosciences, San Jose, CA, USA) was used to detect the stained cells and their cell cycles were evaluated. The Annexin V-FITC/PI apoptosis detection kit was used for Annexin V-FITC/PI analysis.

### 2.7. Analysis of Differentially Expressed Genes

To explore the regulatory mechanism of α-CBD on HepG2 cells at the molecular level, HepG2 cells were treated with 20 mg/L α-CBD for 48 h (experimental group). The total RNA of cells from control and experimental groups was extracted using the Cell RNA Kit (NEB, Ipswich, MA, USA). Subsequently, 5 μg of RNA from each sample was used to construct libraries using the NEB Next Ultra RNA Library Prep Kit from Illumina, according to the manufacturer’s instructions. Differential gene expression analysis between the experimental and the control groups was performed using the DESeq R package (1.18.0). We mainly focused on the function of differentially expressed genes. RNA-Seq data is deposited under the accession codes PRJNA516386, PRJNA516390, PRJNA516391, PRJNA516392, PRJNA516393 and PRJNA516394 in the NCBI SRA database. 

### 2.8. Statistical Analyses

The experiments were performed in triplicates simultaneously. For data analysis, SPSS 21.0 software package (Chicago, IL, USA) was used to determine statistically significant differences between means. Compared with negative controls, differences were considered statistically significant if the *p* value was < 0.05.

## 3. Results

### 3.1. Identification of α-CBD

**Figure 1:**
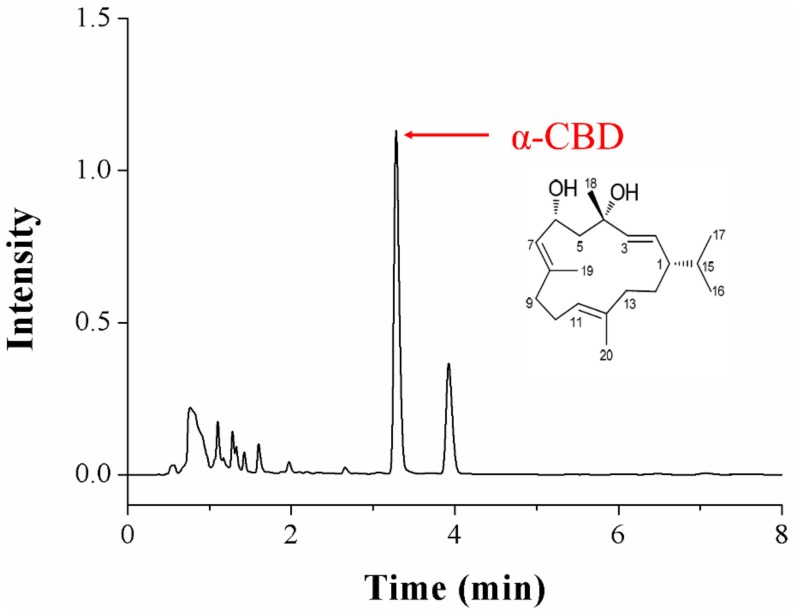
Chromatography results and structure of crude α-2,7,11-cyprotermine-4,6-diol (α-CBD) isolated from *Nicotiana tabacum* L.

The results of the nuclear magnetic resonance spectrum analysis are as follows (Figure 1): HR-ESI-MS *m*/*z*: 329.2460 (M+ Na)^+^, molecular formula is C_20_H_34_O_2_. ^1H^NMR(400 MHz, measured in CDCl_3_) showed: δH 1.72 (1H, m, H-1), 5.36 (1H, dd, *J* = 8.6, 15.7 Hz, H-2), 5.37(1H, m, H-3), 2.00 (1H, m, H-5α), 2.01 (1H, m, H-5β), 4.49 (1H, m, H-6), 5.36 (1H, d, *J* = 8.8 Hz, H-7), 1.51 (1H, m, H-9α), 1.99 (1H, m, H-9β), 2.02(2H, m, H-10), 5.04 (1H, m, H-11), 2.13 (2H, m, H-13), 1.68 (2H, m, H-14), 1.54 (1H, m, H-15),0.83 (3H, d, *J* = 5.8 Hz, H-16), 0.86 (3H, d, *J* = 5.8 Hz, H-17), 1.36 (3H, s, H-18), 1.71 (3H, s, H-19), and 1.56 (3H, s, H-20).^13^C NMR (100 MHz, measured in CDCl_3_) showed: δC 46.43 (C-1, CH), 127.83 (C-2, CH),137.58 (C-3, CH), 72.46 (C-4, C), 52.19 (C-5, CH_2_),66.38 (C-6, CH), 130.61 (C-7, CH), 136.90 (C-8,C), 38.88 (C-9, CH2), 23.34 (C-10, CH_2_), 124.43 (C-11, CH), 133.43 (C-12, C), 36.82 (C-13, CH_2_), 27.97 (C-14, CH_2_), 33.02 (C-15, CH), 19.35 (C-16,CH_3_), 20.66 (C-17, CH_3_), 30.15 (C-18, CH_3_), 16.08 (C-19, CH_3_), and 15.01 (C-20, CH_3_). Therefore, the structure was identified as α-CBD.

### 3.2. α-CBD Reduced the Viability and Prohibited the Proliferation of Hepatocellular Carcinoma Cells

**Figure 2:**
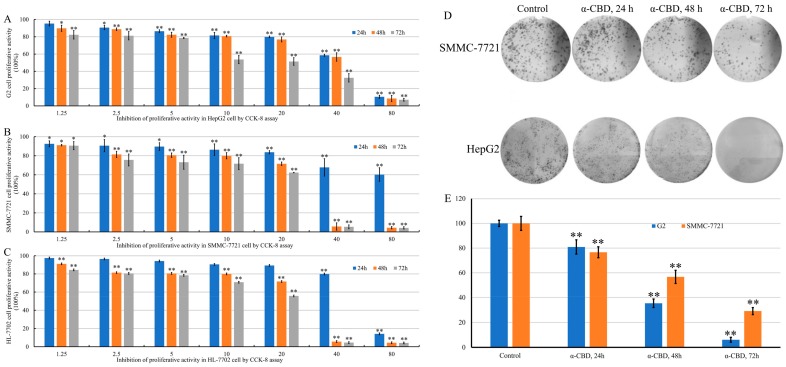
Effect of α-CBD on hepatoma carcinoma cell lines. (**A**) Viability of HepG2 cells after α-CBD treatment, (**B**) viability of SMMC-7721 cells after α-CBD treatment, and (**C**) viability of HL-7702 cells after α-CBD treatment. The survival rate of HepG2 and SMMC-7721 cells after treatment with α-CBD (**D**,**E**) determined by the colony formation assay (*n* = 5; * *p* < 0.05,** *p* < 0.01).

HepG2, SMMC-7721, and HL-7702 cells were treated with different concentrations of α-CBD for 24, 48, and 72 h after the MTT assay. The results revealed that the activity of HepG2 cells in the treated group decreased with time at the same concentration and decreased with increase in concentration at the same time (Figure 2A). The viability of SMMC-7721 cells treated with α-CBD decreased when the concentration was 20–40 mg/L. Negligible changes were observed in the activity of cells treated with 40 and 80 mg/L α-CBD 48 and 72 h (Figure 2B). With the increase in time and concentration, the activity of HL-7702 cells gradually decreased (Figure 2C). After treatment for 24, 48, and 72 h, the activity of HL-7702 cells significantly decreased to almost zero at a concentration of 80 mg/L. In general, the results showed that α-CBD has considerable inhibitory effect on the three types of cells in a time- and concentration-dependent manner. However, the inhibitory effect on HL-7702 was weaker than that on HepG2 and SMMC-7721 cells. HepG2 and SMMC-7721 cells were treated with 20 mg/L α-CBD for 24, 48, and 72 h and the colony formation ability was measured. The colony formation rate of HepG2 and SMMC-7721 was 80.92%, 35.42%, and 6.10% and 76.63%, 56.70%, and 29.13% at 24, 48, and 72 h, respectively. These results show that α-CBD can reduce the colony formation ability and inhibit the proliferation of hepatocellular carcinoma cells.

### 3.3. Effect of α-CBD on the Morphology of Hepatocellular Carcinoma Cells

**Figure 3:**
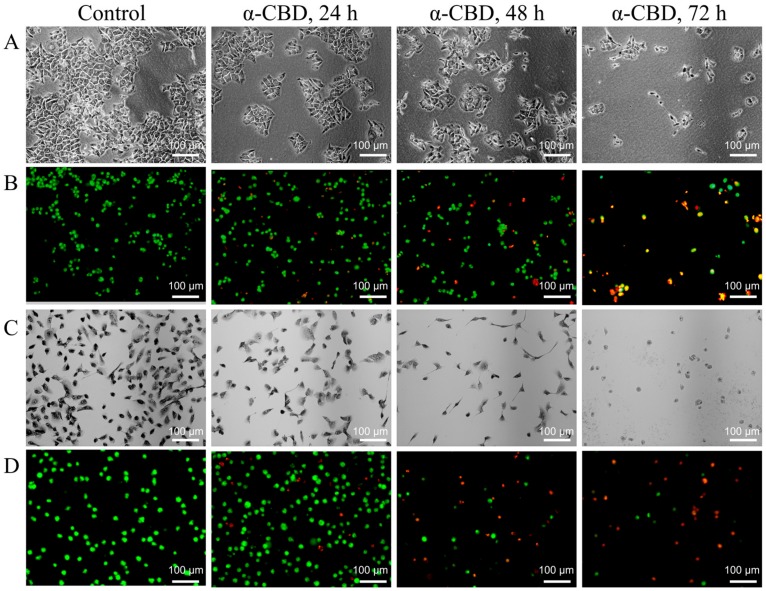
α-CBD induced morphological changes and plasma membrane abnormality in hepatoma carcinoma cells. (**A**) Morphological changes in HepG2 cells after α-CBD treatment; (**B**) AO/EB double-fluorescent staining of HepG2 cells treated with α-CBD; (**C**) Morphological changes in SMMC-7721 cells after α-CBD treatment; and (**D**) AO/EB double-fluorescent staining of SMMC-7721 cells treated with α-CBD.

To explore the effect of α-CBD on the morphology of HepG2 cells, the cells were treated with α-CBD (20 mg/L) for 24, 48, and 72 h. The cells were observed and photographed under an inverted microscope. As shown in Figure 3a, HepG2 cells treated with 20 mg/L α-CBD for different times showed changes in morphology. These changes included shrinkage, roundness, vacuolation, and dissociation from bottle wall. In the control group, HepG2 cells were adherent to the plates, and they were mainly polygonal in shape, and exhibited a high degree of differentiation. By AO/EB fluorescence double staining, we found that cytoplasmic permeability can be improved by α-CBD. The cells of the control group showed uniformed green fluorescence. In the 24 h group, nucleus consolidation was observed, and orange-yellow fluorescence was observed in some nuclei. The cells in the 48h group were slightly round and their edges were not clear. However, some nuclei were stained with orange or orange-red fluorescence. In the 72-h group, the cell density significantly decreased as shown in Figure 3b. SMMC-7721 cells in the control group were large and spindle-shaped, with relatively active nuclear division (Figure 3c). However, the α-CBD-treated groups showed visible morphological changes. With time, the cell density gradually declined, and the cells shrunk. Additionally, the intercellular space was also highly expanded. Up to 72 h, the number of viable cells significantly decreased, and they were smaller and round, and suspended in the culture medium. Furthermore, AO/EB fluorescence double staining of SMMC-7721 cells showed similar changes (Figure 3d).

### 3.4. α-CBD Induced Cell Cycle Arrest and Apoptosis

**Figure 4:**
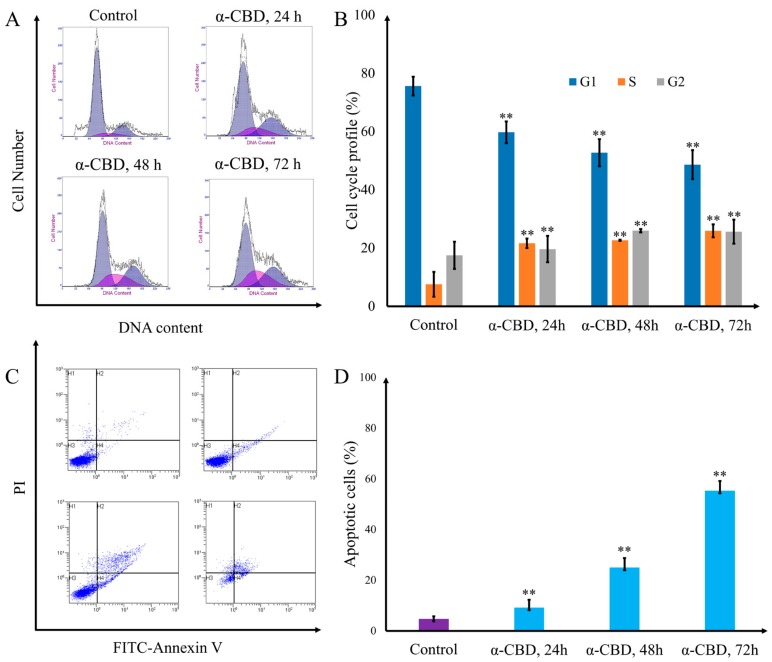
Cell cycle arrest and apoptosis induced by α-CBD in hepatocellular carcinoma cells. (**A**) Patterns of cell cycle distribution in HepG2 cells treated with α-CBD; (**B**) cell cycle profile of CBD-treated HepG2 cells; (**C**) patterns of apoptosis distribution inHepG2 cells treated with α-CBD; and (**D**) apoptotic cells among the total HepG2 cells treated with α-CBD (*n* = 3; ** *p* < 0.01).

HepG2 cells were treated with 20 mg/L α-CBD for 24, 48, and 72 h after flow cytometry, and the number of cells in the S phase was 10.4%, 16.9%, 22.0%, and 29.8%, respectively. The number of cells in the S phase increased significantly, indicating that α-CBD can inhibit the proliferation of hepatocarcinoma cells (Figure 4a,b). The apoptosis rate of HepG2 cells increased with the duration of α-CBD treatment. This showed that α-CBD can induce HepG2 cell apoptosis (Figure 4c,d).

### 3.5. Gene Expression Analysis

**Figure 5:**
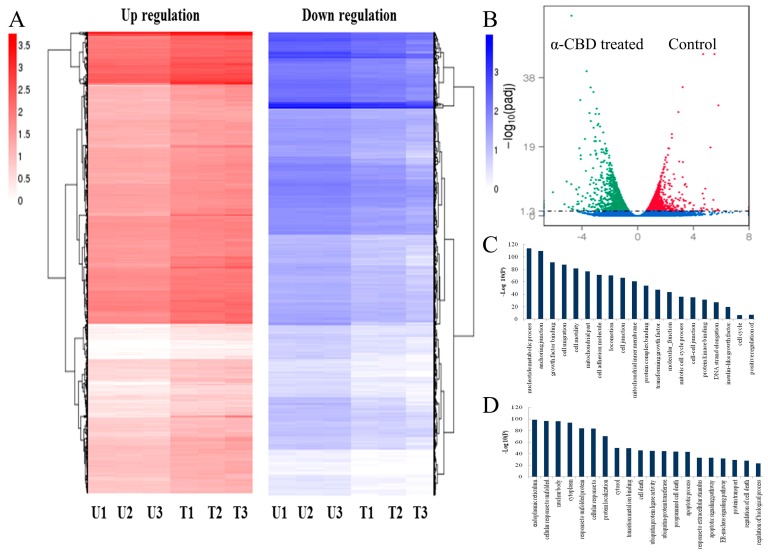
Overview of genes differentially expressed in α-CBD-treated and untreated HepG2 cells. (**A**) Venn diagram of gene expression; (**B**) volcano plot of differentially expressed genes; (**C**) clustering analysis of differentially expressed genes; and (**D**) GO analysis of differentially expressed genes. The control groups are represented as U1, U2, and U3, whereas, the experimental groups are represented as T1, T2, and T3.

The total number of genes expressed in the experimental group was 12,765, of which the number of genes with specific expression was 628. The number of genes expressed in the control group was 12,774, of which the number of genes with specific expression was 637. The number of genes co-expressed in the two groups was 12,137 (Figure 5a). The expression of transcripts in HepG2 cells treated with 20 mg/L α-CBD for 48 h showed that there were 3068 genes with significant differences. Among these genes, the expression of 1289 genes were up-regulated and 1779 were down-regulated (Figure 5b). To further identify the function of genes, we clustered these up-regulated and down-regulated genes and performed the Gene Ontology (GO) and the Kyoto Encyclopedia of Genes and Genomes (KEGG) enrichment analysis (Figure 5c). The differential gene GO enrichment analysis showed that the down-regulated genes mediated cell attachment, cell migration, mitosis, cell cycle, DNA strand extension in DNA replication, and growth factor binding, which inhibit cell survival and proliferation. The up-regulated genes mediated protein localization, metal ion transition, cell death, programmed cell death, apoptosis progression, apoptosis pathway, ubiquitin transferase activity, endoplasmic reticulum signaling pathway, and protein transport, which could promote apoptosis (Figure 5d). The differential gene KEGG pathway enrichment analysis showed that the up-regulated pathways were the NF-κB signaling, HIF-1 signaling, apoptosis, cancer, and autophagy pathways, and the downregulated pathways were adhesion, citric acid cycle, conduction pathway, MAPK signaling pathway, TGF-β signaling pathway, P13K-Akt signaling pathway, and cell cycle. Apoptosis might be mediated by the p53-PUMA, PI3K-Akt, and IL-1-NFkB-IAP pathways (Table 1).

## 4. Discussion

The incidence of hepatocellular carcinoma has gradually increased during recent years [21]. The development of novel therapeutic agents for hepatocellular carcinoma is urgently needed [4,20]. Antimicrobial, antitumor, and neuroprotective activities effects of CBDs have been investigated in previous studies [18,19]. However, the mechanism by which CBDs exerts anticancer effects on hepatocellular carcinoma cells requires further research. Herein, we isolated α-CBD from tobacco and studied its antitumor activity against hepatocellular carcinoma cells by affecting multiple cell signaling molecules based on cytology experiments and a transcriptome profiling study.

The antitumor effect of most anti-tumor drugs is exerted via the inhibition of tumor cells proliferation [22]. The detection of cell proliferation inhibition is often the first step in vitro for antitumor drug development and clinical tumor sensitivity tests. In the present study, the MTT and clone formation assays were used to detect the cell viability and clone inhibition ability of α-CBD, respectively. The MTT test results showed that α-CBD has inhibitory effect on HL-7702, HepG2 and SMMC-7721 cells. However, the inhibitory effect was relatively weak on HL-7702 cells than that on HepG2 and SMMC-7721 cells. Therefore, inhibitory effect was detected only in HepG2 and SMMC-7721 cells. The MTT test also showed that α-CBD can reduce cell viability, with significant effect at a concentration of 20 mg/L. We used the action time as a variable to act on HepG2 and SMMC-7721 cells, and further tested the ability of cell clone formation via the cloning test. The clone formation rate of HepG2 and SMMC-7721 cells gradually decreased with increase in time of action of α-CBD. This suggests that α-CBD can inhibit the clone formation ability of HepG2 and SMMC-7721 cells, thereby, inhibiting cell proliferation. This confirms the results of previous studies that cembratriene-4, 6-diol has antitumor activity [23]. Thus, the effects of α-CBD on HepG2 and SMMC-7721 cells were similar to those of other substances in a cancer cell line [15,24,25]. The above results showed that the substances with antitumor activity similar to that of α-CBD exert antitumor activity by inhibiting the proliferation of tumor cells. Another obvious hallmark of antitumor substances acting on tumor cells is altering the morphology of cells, resulting in apoptosis [26]. One of the main manifestations of apoptosis is morphological changes [27]. In this study, the morphological changes of HepG2 and SMMC-7721 cells treated with α-CBD were observed under a light microscope and a fluorescence microscope. The results showed that the morphology of hepatocellular carcinoma cells and plasma membrane permeability changed, as observed in previous studies [10,13,15,26].

To further explore the mechanism of α-CBD on hepatocellular carcinoma cells, the cell cycle and gene expression in HepG2 cells were detected. The results of flow cytometry demonstrated that α-CBD inhibited the proliferation of hepatocarcinoma cells at the S phase, which is consistent with the results of previous research showing that other antitumor compounds can induce G2/M arrest in human cancer cells [28,29]. Transcriptome profiling demonstrated that these differential genes are related to cell attachment, cell migration, mitosis, cell cycle, DNA strand extension in DNA replication, growth factor binding, protein localization, metal ion transition, cell death, programmed cell death, apoptosis progression, the apoptosis pathway, ubiquitin transferase activity, endoplasmic reticulum signaling pathway, and protein transport, all of which can promote apoptosis. Prior studies have showed that antitumor substances also induce apoptosis by inhibiting the expression of related genes. For instance, Song et al., reported that p53-dependent transcriptional induction of PUMA and oligomerization of Bax played important roles in the sensitivity of cancer cells to apoptosis [30]. Lien et al., reported that the PI3K-Akt pathway plays an important role in tumor cell cycle arrest and apoptosis [31]. Li et al., found that IL-1, NF-κB and IAP are the proteins that inhibit apoptosis and affect the transduction pathway related to survival or cell proliferation [32]. Overall, our data support the conclusion that α-CBD prominently regulated the p53-PUMA, PI3K-Akt, and IL-1-NF-κB-IAP pathways and induced apoptosis. Thus, this study revealed that α-CBD exerted its antitumor effects by affecting cell viability and genome-wide differential gene expression.

## 5. Conclusions

α-CBD from tobacco can decrease the viability of hepatocarcinoma cells, inhibit cell proliferation, alter plasma membrane permeability, promote apoptotic morphology, arrest cell cycle at the S phase, and induce apoptosis through the p53-PUMA, PI3K-Akt, and IL-1-NF-κB-IAP pathways. Applied chemistry should now be used to further enhance the effect of α-CBD on tumor cells through structural modifications. Meanwhile, the relationship between the inhibitory effects and structure-activity of novel antitumor drugs requires a thorough analysis. In addition to providing research basis for screening new anti-tumor drugs, this study also provides technical support for in-depth development and use of active ingredients in tobacco.

## Figures and Tables

**Table 1:** KEGG pathway analysis of differentially expressed genes.

Term	ID	*p*-Value
Upregulated		
NF-kappa B signaling pathway	hsa04064	0.660557
HIF-1 signaling pathway	hsa04066	0.660557
Apoptosis	hsa04210	0.90181
Pathways in cancer	hsa05200	0.90181
Regulation of autophagy	hsa04140	0.938176
Downregulated		
Adherens junction	hsa04520	0.283208
Citrate cycle (TCA cycle)	hsa00020	0.573324
p53 signaling pathway	hsa04115	0.759371
AMPK signaling pathway	hsa04152	0.771113
TGF-beta signaling pathway	hsa04350	0.946339
PI3K-Akt signaling pathway	hsa04151	0.952199
Cell cycle	hsa04110	0.952652

## Supplementary Materials

Supplementary File 1
