# Supplementary figures and images for: Anti-Tumor Activity of Cembranoid-Type Diterpenes Isolated from Nicotiana tabacum L
Source: Biomolecules. 2019 Jan 28;9(2):45. doi: 10.3390/biom9020045 (PMC6406568; doi:10.3390/biom9020045)

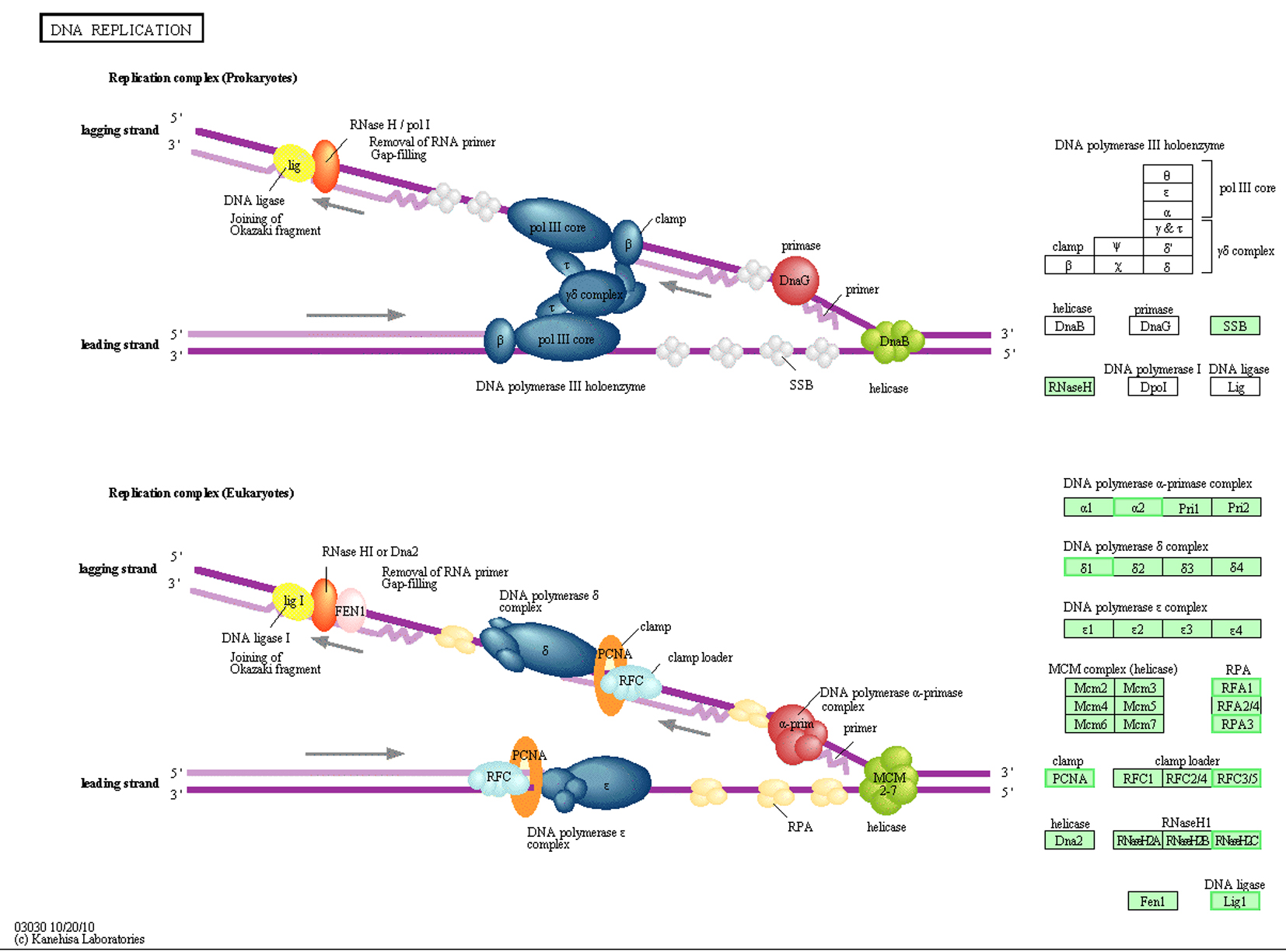

Supplement: Supplementary File 1 [file biomolecules-09-00045-s001.zip › Image_3.JPEG]

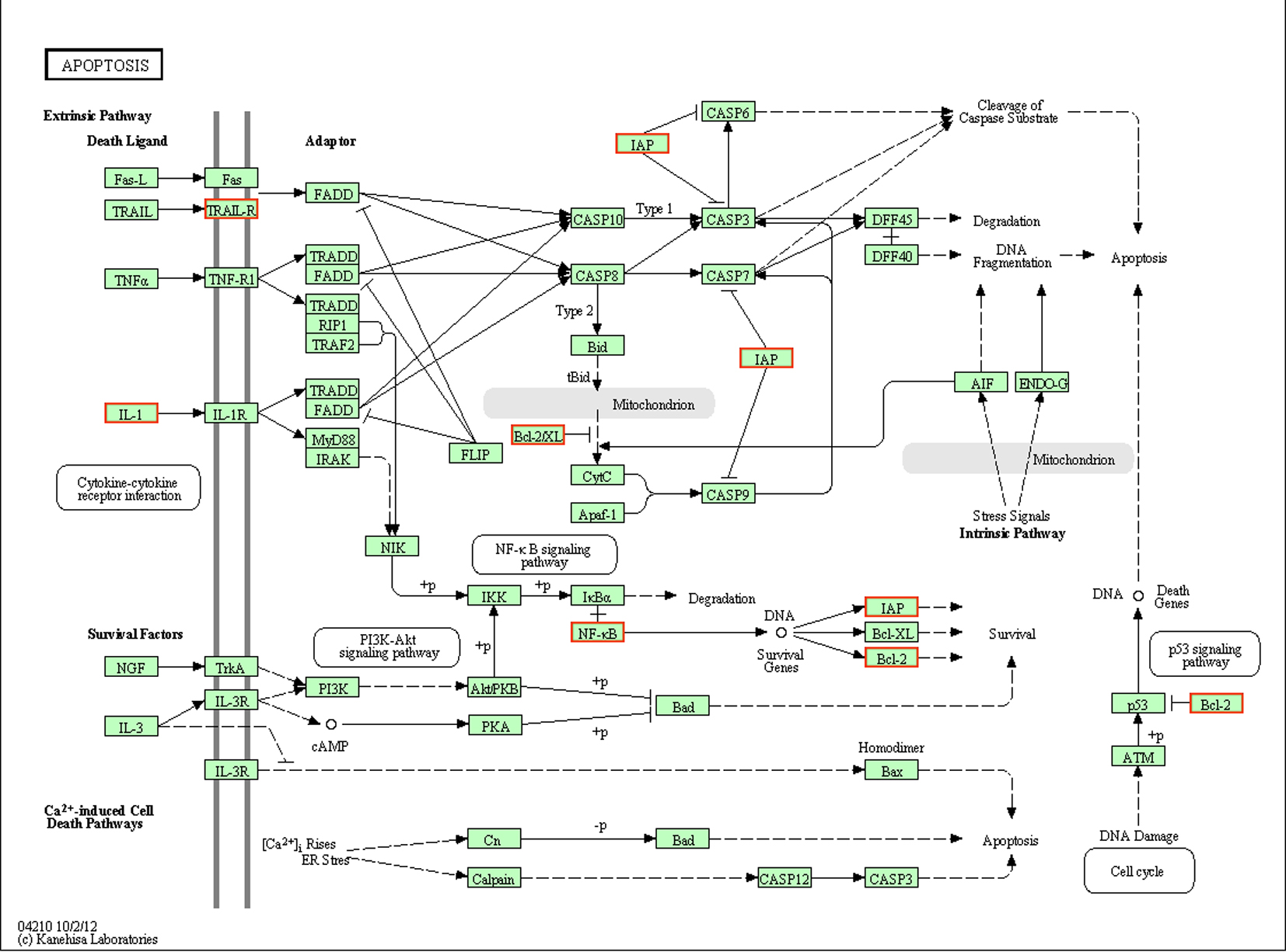

Supplement: Supplementary File 1 [file biomolecules-09-00045-s001.zip › Image_1.JPEG]

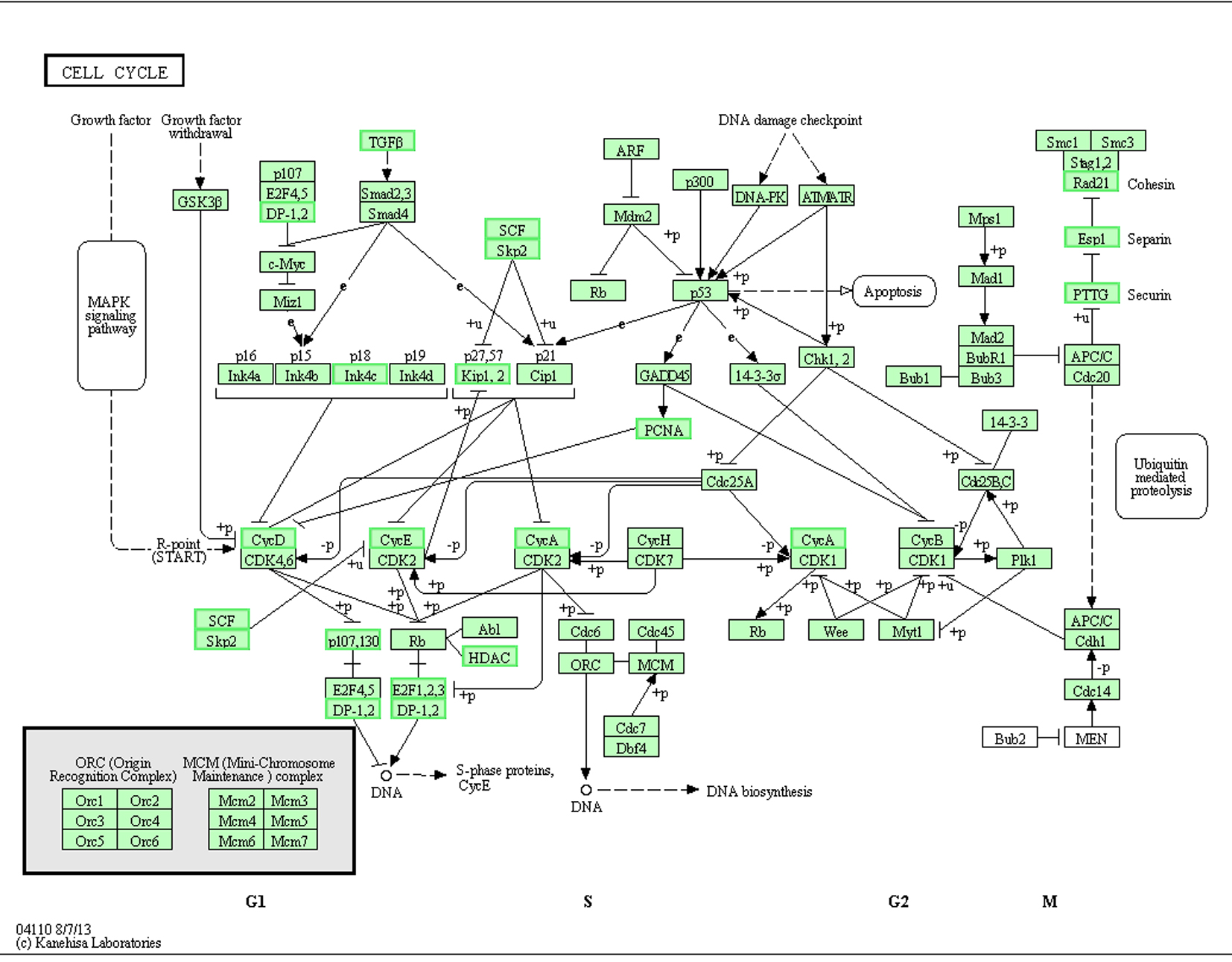

Supplement: Supplementary File 1 [file biomolecules-09-00045-s001.zip › Image_2.JPEG]
